# High spatially sensitive quantitative phase imaging assisted with deep neural network for classification of human spermatozoa under stressed condition

**DOI:** 10.1038/s41598-020-69857-4

**Published:** 2020-08-04

**Authors:** Ankit Butola, Daria Popova, Dilip K. Prasad, Azeem Ahmad, Anowarul Habib, Jean Claude Tinguely, Purusotam Basnet, Ganesh Acharya, Paramasivam Senthilkumaran, Dalip Singh Mehta, Balpreet Singh Ahluwalia

**Affiliations:** 1 Bio-photonics Laboratory, Department of Physics, Indian Institute of Technology Delhi, Hauz-Khas, New Delhi, 110016 India; 2 Department of Physics and Technology, UiT The Arctic University of Norway, Tromsø, Norway; 3 Women’s Health and Perinatology Research Group, Department of Clinical Medicine, UiT The Arctic University of Norway, Tromsø, Norway; 4 Department of Computer Science, UiT The Arctic University of Norway, Tromsø, Norway; 5 Department of Obstetrics and Gynaecology, University Hospital of North Norway, Tromsø, Norway; 6 Department of Clinical Science, Intervention and Technology Karolinska Institutet, Stockholm, Sweden; 7 Department of Physics, Indian Institute of Technology Delhi, Hauz-Khas, New Delhi, 110016 India

**Keywords:** Interference microscopy, Optical imaging, Electrical and electronic engineering

## Abstract

Sperm cell motility and morphology observed under the bright field microscopy are the only criteria for selecting a particular sperm cell during Intracytoplasmic Sperm Injection (ICSI) procedure of Assisted Reproductive Technology (ART). Several factors such as oxidative stress, cryopreservation, heat, smoking and alcohol consumption, are negatively associated with the quality of sperm cell and fertilization potential due to the changing of subcellular structures and functions which are overlooked. However, bright field imaging contrast is insufficient to distinguish tiniest morphological cell features that might influence the fertilizing ability of sperm cell. We developed a partially spatially coherent digital holographic microscope (PSC-DHM) for quantitative phase imaging (QPI) in order to distinguish normal sperm cells from sperm cells under different stress conditions such as cryopreservation, exposure to hydrogen peroxide and ethanol. Phase maps of total 10,163 sperm cells (2,400 control cells, 2,750 spermatozoa after cryopreservation, 2,515 and 2,498 cells under hydrogen peroxide and ethanol respectively) are reconstructed using the data acquired from the PSC-DHM system. Total of seven feedforward deep neural networks (DNN) are employed for the classification of the phase maps for normal and stress affected sperm cells. When validated against the test dataset, the DNN provided an average sensitivity, specificity and accuracy of 85.5%, 94.7% and 85.6%, respectively. The current QPI + DNN framework is applicable for further improving ICSI procedure and the diagnostic efficiency for the classification of semen quality in regard to their fertilization potential and other biomedical applications in general.

## Introduction

Semen quality and male fertility potential have been continuously declining all over the world^1–4^. At the same time, biomedical and technical advances have made it possible to treat male infertility using assisted reproductive technology (ART) including intracytoplasmic sperm injection (ICSI). Evaluation of semen quality and ICSI procedure are the important steps for the successful outcome of ART. Generally, semen parameters evaluation and ICSI procedure are guided by the bright field microscopy and experience of laboratory personnel. Recently, computer-assisted sperm analysis (CASA) a digital microscopic technique made it possible as a machine-based analysis for semen parameters such as sperm cell concentration, sperm cell motility, kinematics, and morphology. For instance, CASA systems acquire successive images of the cells and use special software to track the motion of heads of each spermatozoon^5,6^. However, it fails to provide any supportive information regarding subcellular changes within the sperm cells which could be useful to ICSI procedure. Another powerful technique is a label free holographic imaging, for 3D reconstruction of freely moving sperm cells^7^. Apart from this, other optical as well as spectroscopic techniques have been proposed so far to determine motility of the sperm cells^8–11^. For example, fluorescence imaging and laser scanning confocal microscopy have been demonstrated to investigate the mitochondrial functionality of sperm cells^12^ since the motility of the cells partially depends on the mitochondrial function^13^ and get affected by cryopreservation of the cells. Semen quality is also analyzed based on Raman micro-spectroscopy which can provide the spectral features of human sperm cells^14^. Oxidative stress is also known to affect the integrity of sperm genome, result in lipid peroxidation and decrease in sperm motility, which was quantified recently using partially spatially coherent digital holographic microscopy and machine learning^15^. Additionally, smoking and alcohol consumption are negatively associated with sperm concentration and percentage of motile sperms when compared with the persons without these habits^16^. All these factors can affect the morphology, physiology and subcellular structures of the sperm cells and these changes can be investigated by extracting the quantitative information of the cells.

For label-free sperm imaging, quantitative phase imaging (QPI) is an attractive non-invasive technique to extract the quantitative information of the samples^17–21^. QPI can measure the combined information of refractive index and local thickness of the specimens with a nanometric sensitivity, which can be utilized detecting any deviations from normality^22–24^. QPI has several biological applications, such as 3D imaging of human red blood cells (RBC)^25^, bovine embryo^26^, bovine spermatozoa^27^, tissue imaging^28^, and others. Although, QPI is a potentially powerful technique as it provides morphological changes of the specimens, it has not yet been amenable to interpretation by human experts due to the lack of chemical specificity^29^. Therefore, merging QPI with artificial intelligence (AI) is a promising route to provide virtual image classification of the QPI data^29^. Recently, AI techniques have been used to differentiate between healthy and unhealthy phase images of sperm cells. In these techniques, morphological and texture features are extracted from the head part of the cells, and these features are fed to the machine learning classifier to separate them into diagnostically relevant classes^15,30,31^. However, the variations in an imaging system, and, difficulty in deriving a consistent and reliable feature out of thousands of features, base pose difficulty in applying conventional machine learning techniques for classification of the sperm cells. For example, precise segmentation of the head part is required to measure the length, width and area of the sperm head^30^. Due to the lack of chemical specificity of QPI technique, the boundary between head and mid-piece of the cell cannot be located very precisely. Secondly, measuring the length and area of the head part to differentiate between normal and stressed sperm cells depends on the human expertise and segmentation algorithm. Additionally, the actual value of morphological features such as surface area, volume, surface to volume ratio and sphericity cannot be accurately determined without decoupling the refractive index and thickness of the cells^32^. Therefore, it is beneficial to use advance machine learning technique i.e. convolutional neural network (CNN), which does not require to extract any features and can automatically generate abstract convolutional features from the training dataset. CNN/deep learning is rapidly growing as an automated technique in biomedical imaging for example disease classification^33–35^, image segmentation^36^, resolution enhancement^37^, digital staining^18^, noise reductions^38^, among others^39,40^.

**Figure 1:**
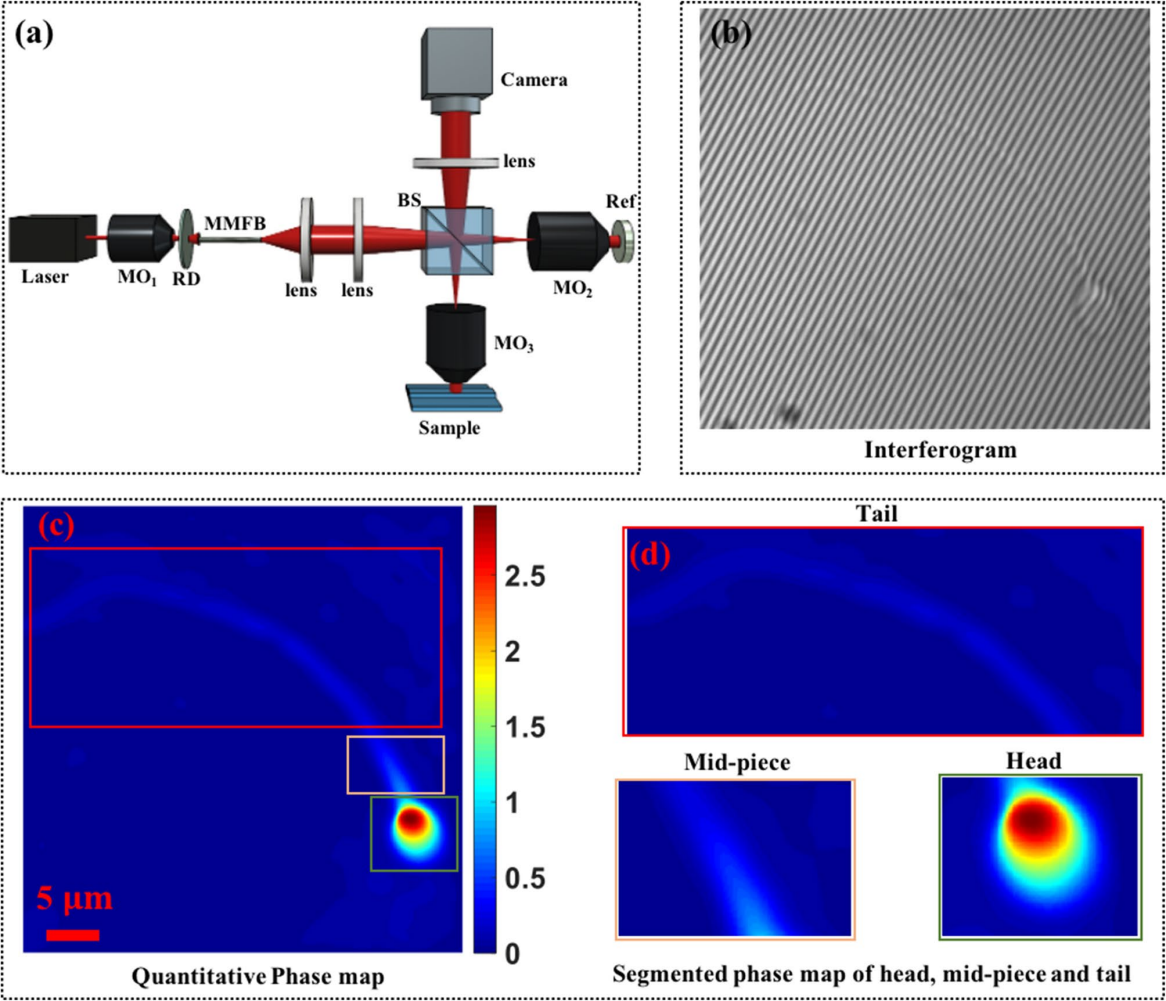
(**a**) Schematic diagram of the partially spatially coherent digital holographic microscope (PSC-DHM) system and (**b**) the interferometric image of the sperm cell acquired from the PSC-DHM. (RD—rotating diffuser, Ref—reference mirror, BS—Beam splitter, MO—microscope objective, MMFB—multiple multi-mode fiber bundle). Reconstructed phase map (**c**) and (**d**) the zoomed view of head, neck and tail part of the sperm cell. Color bar represents the phase map in radian.

We demonstrate the use of QPI technique assisted with deep learning for the classification of sperm cells under different stressed conditions. A total of four different classes were considered in this study, which included healthy, externally induced oxidative stressed, cryopreserved, and externally induced alcohol affected sperm cells. The four classes of sperm cells were also studied using conventional techniques to quantify and compare the progressive and the non-progressive motility of spermatozoa. Figure 1 shows the schematic representation of a partially spatially coherent digital holographic microscope (PSC-DHM) developed to acquire the interferometric images of the sperm cells as can be seen in Fig. 1b. Figure 1c,d shows the quantitative phase map of sperm cells. The PSC-DHM system offers single shot phase reconstruction of the cells especially the thinnest i.e. tail part of the sperms by utilizing partial spatial coherent properties of light source. QPI system commonly uses direct laser light (high spatially and temporally coherent) or white light (spatially and temporally incoherent). Direct laser suffers with low spatial phase sensitivity and hence accurate phase estimation of a thin sample is usually difficult. On the other hand, white light provides high spatial phase sensitivity but due to low temporal coherence it requires phase shifting technique to utilize the whole field of view of the camera. In contrast, the PSC-DHM system offers single shot phase extraction of the sample due to high temporal coherence and high spatial phase sensitivity because of its spatial incoherent nature. The details of the PSC-DHM system is provided in “Methods” section. The spatial phase sensitivity of the system developed is around ± 20 mrad which is utilized to reconstruct the phase information of the cells including the tail part. The thickness of the tail of the sperm cells are typically 100 nm and thus more challenging to image unless high spatial phase sensitivity is achieved. This is particularly useful as the tail plays an important role in progressive motility of the cells and it may change under different stressed conditions.

In this study, total 10,163 interferometric images (2,400 normal, 2,750 cryopreserved, 2,515 oxidative stressed and 2,498 alcohol affected) of the sperm cells were acquired from the PSC-DHM system. The comparison between phase measurement sensitivity of direct laser and PSC source can be found in supplementary information. The phase maps of these cells are used as input to the deep neural network (DNN) which is trained by 70% of the data and validated against the 30% testing data to check the classification accuracy of the networks. The current PSC-DHM + DNN framework has several advantages over the conventional bright field imaging techniques for sperm classification. QPI offers the quantitative information i.e. combined information of the refractive index and the local thickness of the sperm cells. This quantitative morphological information of the sperm cells is not obtained using a bright field microscopy. Additionally, the PSC-DHM system offers a label-free platform with nanometric sensitivity to detect any small sub-cellular changes in the head, mid-piece and tail of the sperm cells. These sub-cellular parts of sperm cells are influenced by the alcohol, oxidative stressed and cryopreservation of the cell. It has been shown that cryopreservation affects the mitochondrial dysfunctionality, damage of cellular membrane, failure of chromatin de-condensation and reduction in motility of the sperm cells^41–45^. Also, oxidative stressed and consumption of alcohol damage the plasma membrane, DNA and reduce the percentage of motile sperm cells^44^. Since, head contain the nucleus i.e. hold DNA of the cells, midpiece packed with mitochondria and tail play an important role in the progressive motility of the cells, therefore, PSC-DHM + DNN could be a valuable label-free tool to detect any morphological changes in these parts of the cells. Finally, DNN architectures provide automated classification of the phase map of the sperm cells. In contrast to the conventional feature extraction-based machine learning classifier^15^, multiple layers in DNN architectures can automatically characterize relevant morphological and texture features to separate the samples into their relevant classes. Therefore, we believe that QPI coupled with DNN would find usage in IVF clinics in diagnosis, and for selection of healthy cells.

## Results and discussion

### Motility test of spermatozoa exposed to ethanol, hydrogen peroxide, and cryopreservation

**Figure 2:**
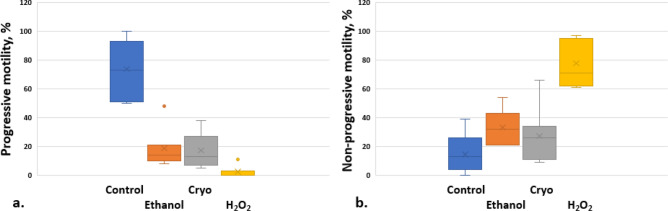
Progressive (**a**) and non-progressive (**b**) motility changes of sperm cells after incubation (1 h/37 °C) with ethanol, hydrogen peroxide (H_2_O_2_) and after cryopreservation as compared with control (n = 7, seven ejaculates from different donors). The middle line of the box represents the median, the “x” represents the mean, the whiskers extend from the ends of the box to the minimum value and maximum value. Outliers marked as dots.

The effect of ethanol, hydrogen peroxide and cryopreservation on progressive and non-progressive motility of spermatozoa was investigated and the results are shown in Fig. 2. Sperm motility were analyzed manually by the trained biologist from the IVF clinic using phase contrast microscope, 40× magnification with Makler counting chamber in accordance as World Health Organization (WHO) standards. Motility are categorized as progressive, non-progressive or immotile. Spermatozoa with progressive motility moves actively, linearly or in a large circle. Non-progressive motility has different patterns of trajectory without progression. The number of progressive motilities assessed first, then the number of non-progressive motility and immortality. Further, progressive and non-progressive motilities are important to count the percentage of motile sperm cells and cross-validate the effect of cryopreservation, externally induced ethanol and H_2_O_2_. After motility test, these sperm cells are imaged by PSC-DHM system for automated identification of normal sperm cells.

In this study, the concentration of H_2_O_2_ and ethanol were selected such that it triggers visible stress effect, i.e. impair the progressive motilities within 1 h. The effect of various concentration of H_2_O_2_ on the motility of sperm cells has been also shown in the previous study^15^. To simulate stress condition, we incubated sperm cells for 1 h in 2% ethanol or 200 µM H_2_O_2_. Lower concentration of ethanol or H_2_O_2_ did not influence the motility strongly in 1 h, at the same time higher concentrations eliminated cells with progressive motility. For cryopreservation, it is assumed that the time of storage semen mammals in liquid nitrogen at − 196 ℃ does not change the viability and the motility of sperm cells. For example, it is shown in the study of Ramírez‐Reveco^46^ that the total sperm motility of bull was not affected by long-term storage at − 196 ℃. In our study thawing of human semen samples were performed in 2–5 h of freezing. Sperm cells were exposed to ethanol or hydrogen peroxide for 1 h at 37 °C. For the control group, same amount of medium as in the test group were added. Figure 2 shows the box plot of the sperm cells after different stressed conditions. After incubation for 1 h, ethanol and H_2_O_2_ produced a significant decrease in progressive motility of sperm cells as compared with control (Table 1): 18.7 ± 13.8% for ethanol and 2.4 ± 4.0% for H_2_O_2_ vs. 73.9 ± 19.5% for control cells. At the same the non-progressive motility after incubation both with ethanol and H_2_O_2_ increased: 33.1 ± 11.9% (ethanol) and 77.7 ± 16.2% (H_2_O_2_) vs. control 14.6 ± 13.8% (ANOVA, paired t-test, p < 0.05). Cryopreservation resulted in a significant decrease in progressive motility (Fig. 2, Table 1). Spermatozoa with progressive motility had a mean value before cryopreservation of 73.9 ± 19.5%, while mean value after thawing was 17.3 ± 11.9% (p < 0.01). The non-progressive motility decreased after thawing, but not significantly: 27.1 ± 19.5% vs. 14.6 ± 13.8% (p > 0.01).

**Table 1:** Effect of cryopreservation, ethanol and hydrogen peroxide incubation on human sperm cells motility.

Variable	Control Mean ± SD	Ethanol, 2% Mean ± SD	Cryopreservation Mean ± SD	H_2_O_2_, 200 µM Mean ± SD	P-value
Progressive motility (PR, %)	73.9 ± 19.5	18.7 ± 13.8	17.3 ± 11.9	2.4 ± 4.0	C/E 0.00009 C/H 0.00005 C/Cryo 0.0001
Non-progressive motility (NP, %)	14.6 ± 13.8	33.1 ± 11.9	27.1 ± 19.5	77.7 ± 16.2	C/E 0.01 C/H 0.0002 C/Cryo 0.2

Analysis of the differences among group means using Paired Two Sample t-Test for Means (alpha 0,05). Values are shown as a mean ± standard deviation (SD). P-value for the analysis of the differences between the sample means of control and ethanol (C/E), control and H_2_O_2_ (C/H), control and cryopreserved groups (C/Cryo).

A possible mechanism of decrease in sperm motility after treatment with ethanol is distortion of cell membrane caused by altering of membrane protein structure^47,48^. On the other hand, the principle mechanism of the effect of hydrogen peroxide on sperm motility is peroxidation of unsaturated fatty acids, which is a part of membrane lipids. As a result of peroxidation, the membrane loses flexibility and plasticity which determines disrupted tail motion^49^. The functional changes of sperm cells after cryopreservation might be influenced by oxidative stress, cryo-protector used (type and concentration), methods of cryopreservation and thawing itself, and is also dependent on original semen characteristics, such as concentration and motility^49^. In accordance with the concept of “partial survival” current procedures used for sperm freezing and thawing lead to decrease of more than 50% in motility and survival rate^50^, ultrastructure and cell morphology changes^51–53^, mitochondrial activity reduction^54^, and damages of sperm chromatin^49^.

**Figure 3:**
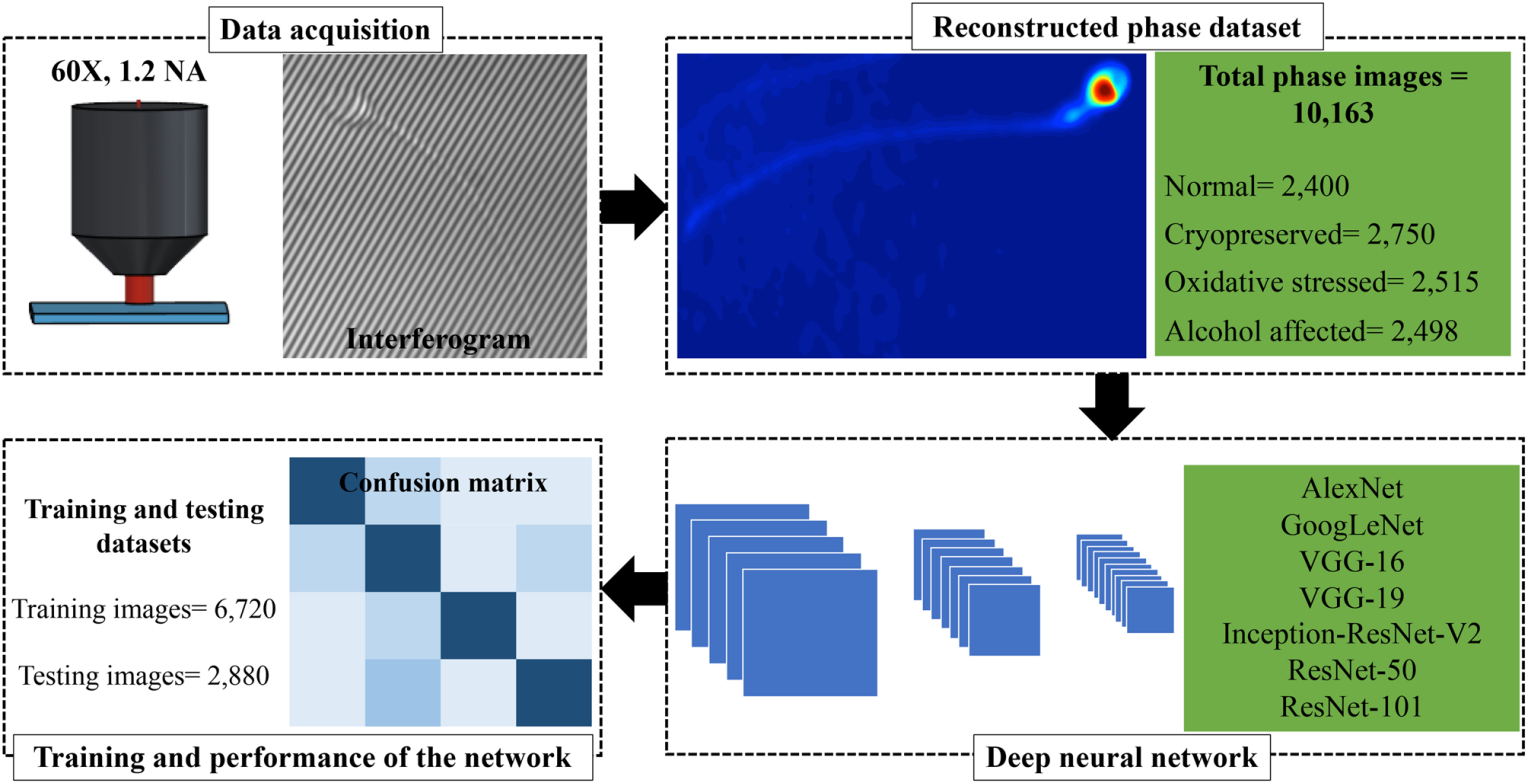
Workflow diagram showing the important steps for the classification of quantitative phase map of sperm cells. Phase map of the images is reconstructed by the interferogram captured using PSC-DHM system. Classification of the phase images is done by total 7 deep neural networks (DNN). Each network is trained with total 6,720 phase images and 2,880 phase images are used to test its accuracy. The final performance and sensitivity of the network is reported in term of confusion matrix.

Figure 3 shows a schematic of our framework for the selection of normal sperm cells. The interferometric image of a sperm cell is acquired from PSC-DHM using 60×, 1.2 NA objective lens. These interferometric images are processed using standard Fourier transform algorithm^55^ and Goldstein phase unwrapping algorithm^56^. The details of the Fourier transform and steps for reconstruction of phase map are described in Methods section. Recently, classification of the phase map of sperm cells is performed using simple machine learning techniques^15^ where texture and morphological features are extracted from the phase image. These features are fed into machine learning models to separate the corresponding phase images into their relevant classes^30^. In contrast, we develop an end-to-end deep learning approach for the classification of normal sperm cells, which does not require extraction of any features from the phase image.

The DNN takes a phase image as input and provide a diagnostically relevant class label as an output i.e. normal cells, H_2_O_2_ stressed cells, ethanol stressed cells, and cryopreserved cells. DNN architecture consists of difference combination of convolution layer, rectifier linear unit layer (ReLU), maxpooling layer, fully connected layer and finally softmax layer. Details of these layers can be found elsewhere^57^. A total of seven DNN, namely AlexNet, GoogLeNet, Inception-ResNet-V2, VGG-16, VGG-19, ResNet-50 and ResNet-101 are used for the classification purpose. These networks are trained by total 70% of the phase images and 30% for testing the accuracy of each model. The training time of the model increased as the number of layers (combination of convolution layer, maxpooling and ReLU layer) increased in the network. The training time and other details of each model is mentioned in the data analysis section. Accuracy of these network are shown in terms of confusion matrices. Confusion matrix shows the number of correct and incorrect prediction of the network. Further, the accuracy of DNN models are compared with total 3 different machine learning classifiers i.e. support vector machine, Naive Bayes and k nearest neighbor. For machine learning classifier, total 11 parameters are extracted from the head part of the cells. These parameters are fed into the classifier for training and testing purpose. The details of all parameters and the classification accuracy of the machine learning models can be found in supplementary information 1.

**Figure 4:**
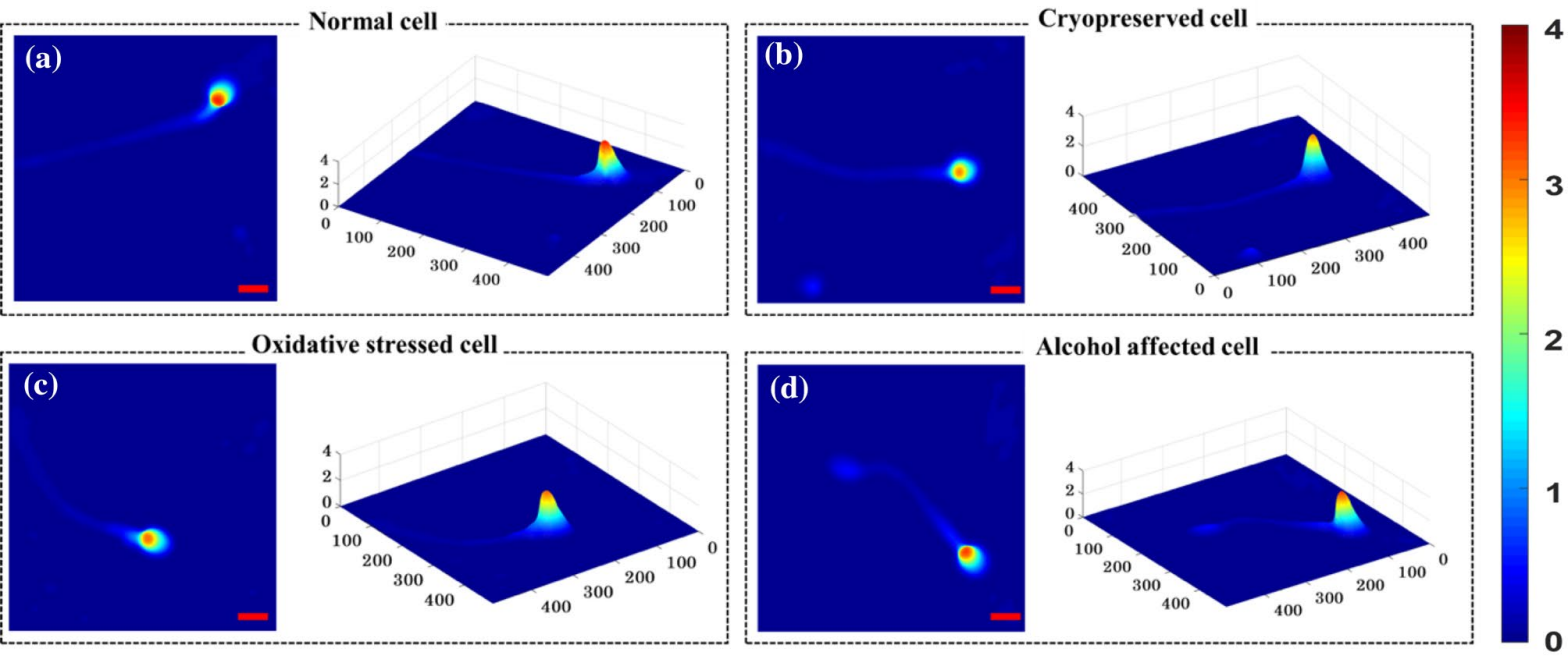
Quantitative phase map of human sperm cells, reconstructed from the interferogram captured by PSC-DHM system: (**a**) normal cell, (**b**) after cryopreservation, (**c**) oxidative stressed cell and (**d**) alcohol affected cell. Color bar represents the phase map in radian. Scale bar: 5 μm.

Figure 4 represents the reconstructed quantitative phase maps of human sperm cells under different stressed conditions. All interferometric images were acquired using 60×, 1.2NA (UPLSAPO 60XW, Olympus) microscopic objective lens. Figure 4a–d are the quantitative phase maps of normal, cryopreserved, externally introduced ethanol and H_2_O_2_ sperm cells respectively. The scale bar corresponds to 5 μm distance in both x and y directions and the color bar shows the phase value in radians. A total 12,332 phase images (2,906 healthy, 2,981 cryopreserved, 3,222 ethanol and 3,223 oxidative stressed) are reconstructed from the interferometric images acquired from the proposed setup. Among the 12,332 images, only those phase images are retained which satisfy the following criteria: correct phase unwrapping, background subtraction and only one cells lies throughout the field of view. The first criterion allows for filtering of phase images that are reconstructed correctly and the later criterion is to promote accurate classification of the sperm cells using DNN. Selection of images with single cells is done automatically by converting phase image into a binary image and setting certain threshold value of white pixels. For the empirical threshold determination, a small set of images with only one cell each were hand-picked as the candidate images. The process of thresholding generally filtered away most images with multiple cells. Nonetheless, all the retained images were checked manually to assess if both the above-mentioned criteria were satisfied, so that the retained images are indeed suitable for classification purpose. A total of 10,163 phase images of sperm cells (2,400 healthy, 2,750 cryopreserved, 2,515 oxidative stressed and 2,498 alcohol affected) are thus retained.

From Fig. 4, the quantitative phase map of healthy sperm’s head is found maximum as compared to the cells under different stressed conditions. The color bar represents the phase map (thickness + refractive index) in radian. Deep red indicates the maximum phase and the deep blue corresponds to zero phase. Change in phase value might indicate the change in morphology of the head of the cells under different stressed condition. However, no general trend in the maximum phase map of the cells is observed which can be explained from the progressive/non-progressive motility and number of mobile (Fig. 2) cells. Although, the number of mobile sperms get decreased as compared to the normal class, there are still some cells which are mobile. Thus, they may sustain their morphology close to normal cell and therefore, no general trend of maximum phase map is observed between these four classes.

**Figure 5:**
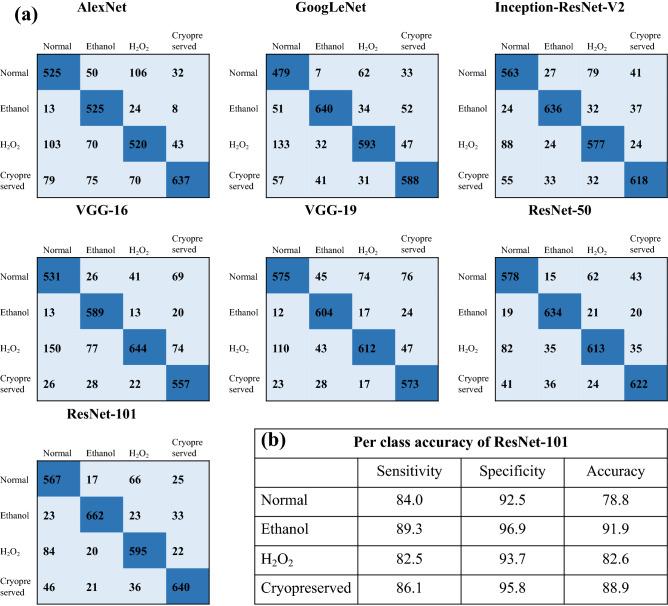
Performance of the deep neural networks (DNN) on the testing datasets of the phase images of sperm cells. (**a**) Confusion matrices of different DNN with number of phase images for classification of healthy and non-healthy phase map of sperm cells. Diagonal elements show number of correct predictions and the off-diagonally elements are the wrong classified observations. (**b**) Per-class sensitivity, specificity and accuracy of ResNet-101.

The performance of the different DNN architectures used in this study are shown in Fig. 5. Total 70% of the data is used for the training purpose while 30% is used to test the accuracy of each model. It is important to note that sufficient training is necessary to train the network and to achieve best accuracy while validating against the test dataset. Figure 5a depicts the confusion matrices of the DNN on the testing datasets of the phase image of sperm cells. The confusion matrices show performance of the network against the testing dataset. Rows and column of the matrix indicate the predicted class and the ground truth respectively. Diagonal elements of the matrix show the correct predictions of the data while off-diagonal elements are the wrong classified data. Consider the confusion matrix of AlexNet in Fig. 5a. Total 720 phase images of the normal phase image of sperm cells are tested by the network. Out of 720, 525 phase images are predicted correctly and 13, 103 and 79 are the wrong predictions. Similarly, 525, 520 and 637 are the correct prediction by the AlexNet for Ethanol, H_2_O_2_ and cryopreserved cells, respectively. Performance of the GoogLeNet, Inception-ResNet-V2, VGG-16, VGG-19, ResNet-50 and ResNet-101 can be also seen in Fig. 5. Note that, the total test images are 30% of the total image (n = 9,600) i.e. 2,880 which is summation of all the elements presents in the matrix.

**Figure 6:**
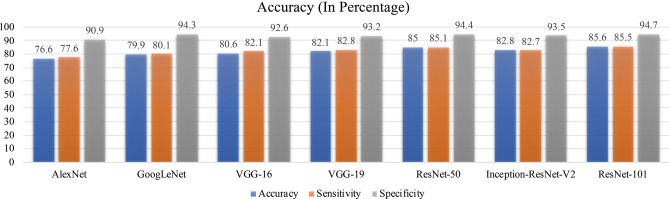
Sensitivity, specificity and classification accuracy of different deep neural network. The blue bar shows the average accuracy of each architectures out of which ResNet-101 provide the best accuracy (85.6%) on the testing datasets.

Figure 6 depicts the average sensitivity, specificity and classification accuracy of all deep learning architectures. Out of all DNN, ResNet-101 provides the best sensitivity, specificity and accuracy of 85.5%, 94.7%, and 85.6% respectively. Final value for ResNet-101 represents the average value of sensitivity, specificity and accuracy shown in the table of Fig. 5b. The training time for each architecture is shown in data analysis section. Though, performance of DNN classifiers is much better than the feature extraction-based machine learning classifier for the same datasets, the mismatch between ground truth and prediction of the network can be understand with following reasons. QPI offers optical thickness i.e. combine information of refractive index and thickness of the specimens but due to the lack of chemical specificity, it cannot precisely identify the changes in different organelles such as mitochondria, nucleus, DNA and the membrane of the cells. Therefore, a label free technique with high chemical specificity is required for more accurate classification of the sperm cells. Additionally, the technique must offer the quantitative changes in the sample under different stressed condition. Virtual staining^18^ in the phase map of sperm cells using deep learning can improve the chemical specificity of the QPI technique. Virtual staining in the phase imaging can offer the chemical changes in the cells under different stressed condition and simultaneously the morphological changes in the cells. Therefore, combining stained information with QPI technique might improve the classification accuracy of the system.

However, a careful examination must be required during the training process and to locate the stained part i.e. head, mid-piece and tail of the cells. Any discrepancies in the training set i.e. mismatch in the correlation between stained mid-piece and phase map of mid-piece can degrade the final performance of the network. Finally, PSC-DHM can be replace with phase shifting low coherence interferometry (PS-LCI) technique^58^. PS-LCI requires multiple frames but offer superior phase sensitivity as well as lateral resolution as compare to other QPI techniques. Using, PS-LCI might help to detect the fine structural changes in the sperm cells thus will be useful to detect the healthy sperm cells with better accuracy. Nonetheless, PS-LCI requires multiple frames to extract the phase map of the cells whereas PSC-DHM can extract the phase map with a single frame with high spatial phase sensitivity.

## Conclusion

Our current QPI + DNN framework allows for the automated classification between normal and abnormal sperm cells. QPI is a promising technique which provides quantitative information of the sample and hence an edge over the conventional intensity-based identification of healthy cells. PSC-DHM system has been used to extract the quantitative phase map of the sperm cells enabling single shot phase reconstruction with high spatial phase sensitivity (± 20 mrad). High spatial phase sensitivity is utilized to acquire the phase map of the entire sperm cells, i.e. head, neck and tail of the sperm cells, which is otherwise difficult to image using direct laser based DHM^15^. Sperm cells after cryopreservation, oxidative stress and exposure to ethanol are imaged by the proposed setup. Previous studies show that oxidative stress initiates concentration dependent increase of DNA fragmentation because of DNA strand breaks^47^. Also, ethanol distorted the cell membrane resulting from the alteration of membrane protein structure^4^. Therefore label-free, non-invasive methods such as PSC-DHM are highly desirable to detect these changes and hence for the selection of good quality sperm for ICSI procedure that can be used to improve the success of ART however it need further clinical trials.

Quantitative analysis of sperm cells provides an opportunity to identify healthy sperm cells using deep learning approaches. Deep learning can be potentially a powerful technique for automated classification of sperm cells into normal and abnormal. Our results demonstrate that a variety of DNN architectures provide good classification accuracy to separate the 4 different type of sperm cells into their relevant classes. We also compared the proposed method with previously shown feature extraction-based machine learning models for the classification of sperm cells. However, these classifiers (SVM, Naive Bayes and KNN) provide very poor accuracy as compare to the modern DNN where ResNet-101 provided the best accuracy, i.e. 85.6%, for classification into healthy, oxidative stressed, cryopreserved and ethanol affected sperm cells. Moreover, the use of seven different network allows us to understand the capabilities of each networks and to apply best deep learning architecture for the identification of healthy cells. We applied our automated classification model for studying clinically relevant problems of semen quality in different patients attending the in-vitro fertilization clinic of University Hospital of North Norway, Norway. Fully automated classification of the sperm cells could be an intermediate tool for the expert that can be utilized for the selection of healthy sperm cells as per World Health Organization (WHO) criteria^59^. QPI + DNN framework for healthy sperm identification could be potentially used for real time selection of healthy living sperm cells that can be used for improving the success of fertilization during ART procedures.

## Methods

### Experimental setup

PSC-DHM is developed for quantitative phase imaging (QPI) of the sperm cells. Schematic diagram of PSC-DHM setup is shown in Fig. 1. To reduce the spatial coherence of the direct laser (He–Ne laser), it first focused by using microscopic objective lens (MO_1_) and rotating diffuser is placed at the focus plane of the MO_1_. Rotating diffuser scattered the light into multiple directions which captured by multi-multimode fiber bundle (MMFB). Output of MMFB consist high temporally and low spatial coherence properties and thus act as an extended light source. The extended light source coupled at an input port of the Linnik type interferometer. The light beam is first collimated and then focused by using a combination of lens L_1_ (f = 50 mm) and L_2_ (f = 150 mm) respectively. A beam splitter is placed to divide the focused beam into reference and sample. The sample beam is focused into the back focal plane of MO_3_ (UPLSAPO 60XW, Olympus) and hence the output beam is nearly collimated to extract the accurate phase information of the sample. The light beam reflected from the sample and reference mirror, interfere at the beam splitter plane which consist the coded phase information of the sample. The interferogram is finally projected into the camera sensor (Hamamatsu ORCA-Flash4.0 LT, C11440-42U) by using a tube lens L_4_ (f = 150 mm). The 2D intensity variation of an interferogram can be expressed as:

1 $$I\left(x,y\right)=a\left(x,y\right)+b\left(x,y\right)cos\left[2\pi i{(f}_{x}x+{f}_{y}y)+\phi \left(x,y\right)\right]$$

where ${f}_{x}$ and ${f}_{y}$ are the spatial carrier frequencies of the interferogram. Background (DC) and the modulation terms are defined by $a\left(x,y\right)$ and $b\left(x,y\right)$, respectively. Phase $\phi \left(x,y\right)$ contains information of the specimen. By applying Fourier transform, the phase map of the specimen can be measured by the following expression:

2 $$\phi (x,y)={\tan}^{-1}\left[\frac{Im\left(c\left(x,y\right)\right)}{Re\left(c\left(x,y\right)\right)}\right]$$

where

3 $$c\left(x,y\right)=b\left(x,y\right)exp\left(i\phi (x,y)\right)$$

where Im and Re are the imaginary and real part of the complex signal. As the wrapped phase map lies between –π to + π, the unwrapping is done by standard Goldstein phase unwrapping algorithm^56^.

### Semen preparation

The Regional Committee for Medical and Health Research Ethics of Norway (REK_nord) has approved the study. Ethical guideline was followed. At the IVF Clinic, Department of Obstetrics and Gynecology, University Hospital North Norway, Tromsø, 7 semen samples were collected from patients who were attended the IVF clinic for the service of ART. The sample were collected from the patients of age between 30 and 40 years. All patients were informed, and informed consent was obtained. The semen sample was collected according to the criteria established by the WHO^59^ after 3–5 days of assistance. After liquefaction, sperm counts were evaluated using the Neubauer-improved counting chamber. All ejaculates used in the experiments meet as the good quality semen sample as per requirements of WHO 2010 (Table 2). To eliminate seminal plasma and isolate cells with good quality sperm cells, one milliliter of semen was carefully placed on each 1.5 ml of 90% and 45% gradient layers (Vitrolife, Sweeden) and centrifugated at 500*g* for 20 min. The resultant pellet was washed twice with human Quinn’s sperm washing medium (Origio, Denmark) at 300 g for 10 min. The supernatant was discarded, and the concentration of the cells from the pellet was adjusted to 1 × 10^6^ sperm per mL with Quinn’s Advantage fertilization medium (Origio, Denmark) supplemented with 5 mg/ml Human Serum Albumin (Sigma).

**Table 2:** Age and semen quality measured before the purification by gradient method (n = 7, number of donors).

Parameter	Mean ± SD
Age (years)	34.7 ± 4.8
Semen volume (ml)	3.1 ± 1.5
Semen concentration (× 10^6^/ml)	51.6 ± 22.8
Total sperm count (× 10^6^)	166.8 ± 142.1
Progressive motility (%)	59 ± 11.9

Values are shown as a mean ± standard deviation (SD).

To perform experiments, 96-well cell culture plates (Corning) were filled with purified sperm in a concentration of 2 × 10^4^ cells per mL with 200 µM H_2_O_2_ (for oxidative stressed samples) or 2% ethanol (for alcohol affected samples), the reference chamber was filled with purified semen only. The samples were incubated for 1 h at 37 °C, 5% CO_2_. After incubation evaluation of cell motility was graded according to WHO 2010 criteria as a progressive (PR) and non-progressive motility (NP). Sperm counting was performed using Neubauer-improved counting chamber and examined under the inverted phase contrast microscope at 40× magnification. Cryopreservation and thawing of purified semen were performed in accordance with Sperm Freeze medium protocol (Origio, Denmark). Motility of post-thaw spermatozoa was evaluated using Neubauer-improved counting chamber. For the purpose of quantitative analysis by PSC-DHM the cells of each sample were transferred in a PDMS chamber on reflecting silicon. Sperm cells were immobilized by fixation with 4% PFA for 30 min at RT and washed in PBS (Phosphate-Buffer Saline, Sigma) for 5 min. Finally, fixed and attached at the surface of PDMS chamber sperm cells were mounted in PBS and covered by the cover glass of 170 μm thickness.

### Data analysis

Extracting phase information from the interferogram and deep learning is implemented in MATLAB 2019a on a 64-bit Windows OS, Intel Xeon CPU E5-1650 v4 @ 3.6 GHz with 64 GB RAM and NVIDIA 2080 Ti GPU. Transfer learning is performed by using pretrained DNN and retraining them for our desired classes. Classification results are obtained by randomly assigning 70% of the images in the dataset for training and the remaining 30% for testing. The training time of each DNN can be seen in Table 3. In each training iteration, the initial learning rate is set as 10-4 and stochastic gradient descent with momentum (SGDM) is used for training. Maximum number of epochs in the learning process is set as 30.

**Table 3:** Training time of total 7 deep neural network for the classification of normal and stressed affected phase map of sperm cell.

Deep neural network for classification of sperm cells	Training time (s)
AlexNet	3,222
GoogLeNet	8,359
Inception-ResNet-V2	60,992
VGG-16	5,183
VGG-19	8,783
ResNet-50	7,756
ResNet-101	16,943

## Supplementary information

Supplementary information
