# Supplementary material for: High spatially sensitive quantitative phase imaging assisted with deep neural network for classification of human spermatozoa under stressed condition
Source: Sci Rep. 2020 Aug 4;10:13118. doi: 10.1038/s41598-020-69857-4 (PMC7403412; doi:10.1038/s41598-020-69857-4)
Supplement: Supplementary file 1 — Supplementary information [file 41598_2020_69857_MOESM1_ESM.docx]

**High spatially sensitive quantitative phase imaging assisted with deep neural network for classification of human spermatozoa under stressed condition**

Ankit Butola^1, 2#^, Daria Popova^2, 3#^, Dilip K Prasad^4^, Azeem Ahmad^2^, Anowarul Habib^2^, Jean Claude Tinguely^2^, Purusotam Basnet^3,5^, Ganesh Acharya^5,6^, Paramasivam Senthilkumaran^7^, Dalip Singh Mehta^1, 7^ and Balpreet Singh Ahluwalia^2,6^

**Supplementary information**

**Quantitative phase imaging using partially spatially coherent digital holography microscopy**

The interferometric images of the sperm cells are recorded using partially spatially coherent digital holography microscopy (PSC-DHM) system. These images are reconstructed using standard Fourier transform (FT) algorithm^1^ and Goldstein phase unwrapping algorithm^2^. Figure 1 depicts the phase reconstruction comparison between the direct laser and partially spatially coherent light source. Speckles and spurious fringe pattern degrade the quality of interferogram (see Fig. 1 (a)) thus, poor spatial phase sensitivity (±200 mrad) observed using direct laser (Fig. 1(c)). Spatial phase sensitivity relates with phase measurement accuracy of the system i.e. larger the value, poorer will be the accuracy. Therefore, the thinnest part of the sperm cells i.e. tail part cannot be clearly seen with direct laser illumination. Additionally, spatial phase sensitivity in case of partially spatially coherent light illumination (Fig. 1 (d-f)) is found 10 times higher than the direct laser and hence phase reconstruction of thinnest part of the sperm i.e. tail can be clearly visible in Fig. 1 (e).


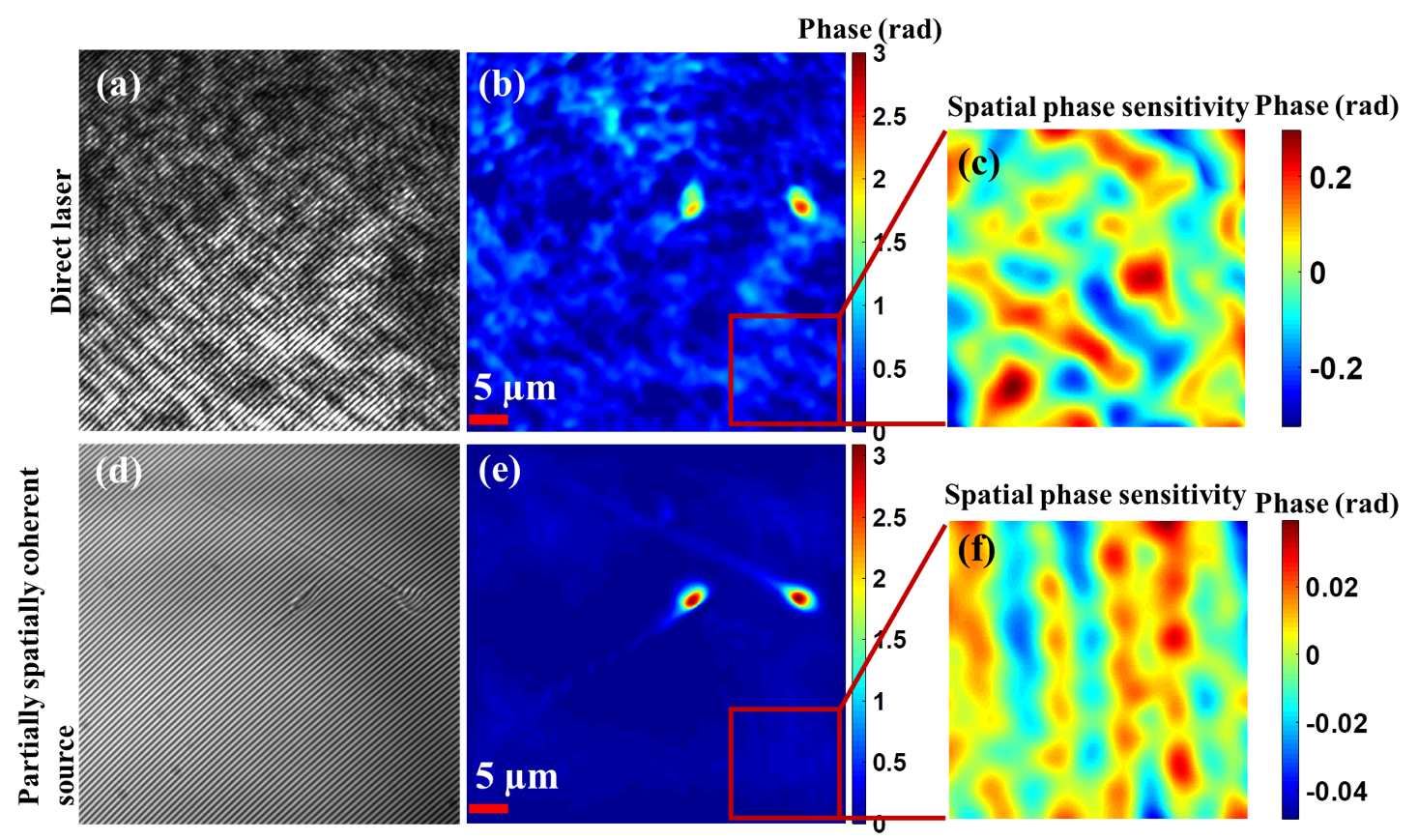


**Figure 1:** Comparison between direct laser and partially spatially coherent light source for the quantitative phase imaging of sperm cells. (a) and (b) represents the interferogram and reconstructed phase map of the sperm cells using direct laser. Figure (d) and (e) shows the interferogram and phase map of same location using partially spatially coherent light source where mid-piece and tail part are clearly visible. The spatial phase sensitivity of the direct laser and partially spatially coherent source are shown in (c) and (f), respectively. Spatial phase sensitivity represents the phase noise presents in the system.

**Classification of sperm cells using machine learning models**

Here, we compared the performance of DNN with previously suggested feature extraction machine learning models^3,4^. Recent studies shown the feature extraction methods for the classification of sperm cells. These feature are extracted from the head part of the sperm cells^3,4^ to differentiate between healthy and unhealthy sperm cells. In this study, total 11 features i.e. mean, variance, energy, entropy, skewness, kurtosis, optical thickness, surface area, volume, surface to volume ratio and sphericity are extracted from 9600 phase images (2400 normal, 2400 Ethanol, 2400 H_2_O_2_ and 2400 Cryopreserved) of head part of the sperm cells. The feature formula^3-5^ and their normalized median ± standard deviation value for each class of the sperm cells can be seen in Table 1.

Further, 3 different machine learning classifiers i.e. SVM, Naive Bayes and KNN are used for the comparison purpose. Statistics and Machine Learning Toolbox of MATLAB 2019a is used for data interpretation. K-fold cross validation is done 5 times to improve the robustness the model. Figure 2 depicts the confusion matrix and receiver operating characteristics (ROC) curve for SVM, Naive Bayes and KNN model. The diagonal elements of the confusion matrix show the correct prediction while off-diagonal corresponds to the incorrect prediction. Additionally, the ROC curve represents the accuracy of the model and plotted between normal and other classes of sperm cells. X and Y-axis of the ROC curve shows false positive and true positive rate, respectively. It is found that the SVM, Naive Bayes and KNN model provide the classification accuracy of 39.1%, 36.4% and 37.5% respectively.

**Table 1**: Morphological and texture features extracted from the sperm head cells for normal, ethanol, H_2_O_2_ and cryopreserved sperm cells. Normalized median ±standard deviation value of total 9600 sperm head cells (2400 normal, 2400 Ethanol, 2400 H_2_O_2_ and 2400 Cryopreserved) are shown here.

| Feature | Formula | Normalized (median ±standard deviation) value of the feature | | | |
| --- | --- | --- | --- | --- | --- |
|  |  | Normal | Ethanol | H_2_O_2_ | Cryopreserved |
| Surface area (S) | $\iint\sqrt{1+g_{x}+g_{y}} dxdy$ | 0.37±0.09 | 0.39±0.08 | 0.37±0.09 | 0.35±0.06 |
| Volume (V) | $\iint OT(x,y) dxdy$ | 0.24±0.10 | 0.25±0.10 | 0.24±0.11 | 0.20±0.07 |
| S/V | $S/V$ | 0.22±0.08 | 0.22±0.07 | 0.21±0.08 | 0.25±0.06 |
| Sphericity | $\frac{\left( 4.84\times V^{\frac{2}{3}} \right)}{S}$ | 0.68±0.08 | 0.67±0.08 | 0.69±0.08 | 0.65±0.07 |
| Optical thickness (OT) | $\phi(x,y)\times\lambda/4\pi$ | 0.40±0.07 | 0.39±0.07 | 0.41±0.07 | 0.39±0.08 |
| Mean () | $\frac{1}{N}\frac{1}{M} {}_{i=1}^{N}{}_{j=1}^{M}\phi_{ij}$ | 0.35±0.06 | 0.36±0.05 | 0.34±0.06 | 0.32±0.04 |
| Variance(${}^{2}$) | $\frac{1}{N} {}_{i=1}^{N}{}_{j=1}^{M}\left( \phi_{ij}- \right)^{2}$ | 0.31±0.06 | 0.30±0.06 | 0.32±0.06 | 0.28±0.05 |
| Energy | ${}_{i, j=0}^{N-1}\left( \phi_{ij} \right)^{2}$ | 0.35±0.06 | 0.36±0.05 | 0.35±0.06 | 0.33±0.04 |
| Entropy | $-{}_{i=1}^{N}p\left( \phi_{i} \right) {log}_{2}p\left( \phi_{i} \right)$ | 0.31±0.08 | 0.33±0.08 | 0.31±0.08 | 0.30±0.06 |
| Skewness | $\frac{1}{N} {}_{i=1}^{N}{}_{j=1}^{M} \left[ \frac{\left( \phi_{ij}- \right)}{} \right]^{3}$ | 0.37±0.15 | 0.37±0.15 | 0.36±0.15 | 0.36±0.14 |
| Kurtosis | $\frac{1}{N} {}_{i=1}^{N} {}_{j=1}^{M}\left[ \frac{\left( \phi_{ij}- \right)}{} \right]^{4}-3$ | 0.25±0.15 | 0.25±0.14 | 0.24±0.15 | 0.24±0.13 |

The true positive rate (sensitivity), true negative rate (specificity) and false positive rate (1-specificity) of the classifier can be defined as

$$True positive rate =\frac{True positive}{True positive+False negative}$$

$$True negative rate = \frac{True negative}{True negative+False positive}$$

$$False positive rate = \frac{False positive}{True negative+False positive}$$


**Figure 2:** Evaluation of the performance of machine learning models for classification of 9600 phase images (2400 normal, 2400 Ethanol, 2400 H_2_O_2_ and 2400 Cryopreserved) of head part of the sperm cells. Confusion matrix and receiver operating characteristic (ROC) curve shows the classification performance and final accuracy of support vector machine, Naive Bayes and KNN model. SVM, Naïve Bayes and KNN model provide the classification accuracy of 39.1%, 36.4% and 37.5% respectively.

The poor classification accuracy of these model can be explained by increasing the number of classes in the datasets. Increasing the number of classes and phase images makes the datasets very complex to handle with simple machine learning models. The extraction of relevant morphological and texture features will certainly affect the final accuracy of the model. Since, the diversity of diagnostic features, variation in imaging system such as illumination and numerical aperture (NA) dependent resolution, and, most importantly, difficulty in deriving a consistent feature create difficulty in applying conventional feature extraction machine learning techniques for classification of the cells. For example, precise segmentation of the head part is required to measure the length, width and area of the sperm head^4^. However, due to the lack of chemical specificity of QPI technique, the boundary between head and mid-piece of the cell cannot be located very precisely. Secondly, measuring the length, width and area of head part to differentiate between normal, alcohol affected and cryopreserved sperm cells depends on the human expertise and segmentation algorithm. Additionally, the actual value of morphological features such as surface area, volume, surface to volume ratio and sphericity cannot be accurately determined without decoupling the refractive index and thickness of the cells^5^.

On the other hand, not only the head but various organelles in sperm cells affected due to the negative effect of cryopreservation, oxidative stressed and alcohol consumption. For example, cryopreservation affects the mitochondrial dysfunctionality, damage of cellular membrane, failure of chromatin de-condensation and reduction in motility of the sperm cells^6-10^. Also, oxidative stressed and consumption of alcohol damage the plasma membrane, DNA and reduce the percentage of motile sperm cells^9^. Since, head contain the nucleus i.e. hold DNA of the cells, midpiece packed with mitochondria and tail play an important role in the progressive motility of the cells, therefore, extracting features only from the head part of the cells cannot offer accurate classification between healthy and stressed sperm cells.

Therefore, it is recommended to use modern DNN architectures which essentially support abstract characterization of all the physical and relevant statistics to these features. Also, it does not require segmentation of head part and utilize morphology of all parts i.e. head, midpiece and tail of the sperm cells to detect the changes in phase value. It can be seen in the manuscript that different DNN architectures are providing good classification accuracy and 85.6% is the best accuracy achieved by ResNet-101 network for the similar datasets. Therefore, PSC-DHM+DNN provides an advance and more robust platform for label-free classification of normal, cryopreserved, oxidative stressed and ethanol affected sperm cells.

**References:**

1 Takeda, M., Ina, H. & Kobayashi, S. Fourier-transform method of fringe-pattern analysis for computer-based topography and interferometry. *JOSA* **72**, 156-160 (1982).

2 Goldstein, R. M., Zebker, H. A. & Werner, C. L. Satellite radar interferometry: Two-dimensional phase unwrapping. *Radio science* **23**, 713-720 (1988).

3 Dubey, V. *et al.* Partially spatially coherent digital holographic microscopy and machine learning for quantitative analysis of human spermatozoa under oxidative stress condition. *Scientific reports* **9**, 1-10 (2019).

4 Mirsky, S. K., Barnea, I., Levi, M., Greenspan, H. & Shaked, N. T. Automated analysis of individual sperm cells using stain‐free interferometric phase microscopy and machine learning. *Cytometry Part A* **91**, 893-900 (2017).

5 Girshovitz, P. & Shaked, N. T. Generalized cell morphological parameters based on interferometric phase microscopy and their application to cell life cycle characterization. *Biomedical optics express* **3**, 1757-1773 (2012).

6 Chaveiro, A., Machado, L., Frijters, A., Engel, B. & Woelders, H. Improvement of parameters of freezing medium and freezing protocol for bull sperm using two osmotic supports. *J Theriogenology* **65**, 1875-1890 (2006).

7 Watson, P. F. The causes of reduced fertility with cryopreserved semen. *Animal reproduction science* **60**, 481-492 (2000).

8 Wongtawan, T., Saravia, F., Wallgren, M., Caballero, I. & Rodríguez-Martínez, H. Fertility after deep intra-uterine artificial insemination of concentrated low-volume boar semen doses. *Theriogenology* **65**, 773-787 (2006).

9 Agarwal, A., Prabakaran, S. A. & Said, T. M. Prevention of oxidative stress injury to sperm. *Journal of andrology* **26**, 654-660 (2005).

10 Lemma, A. Effect of cryopreservation on sperm quality and fertility. *Artificial insemination in farm animals* **12**, 191-216 (2011).
